# Is the Tolerance of Commercial Peach Cultivars to Brown Rot Caused by *Monilinia laxa* Modulated by its Antioxidant Content?

**DOI:** 10.3390/plants9050589

**Published:** 2020-05-05

**Authors:** Vitus I. Obi, Joaquín Montenegro, Juan J. Barriuso, Fayza Saidani, Christophe Aubert, Yolanda Gogorcena

**Affiliations:** 1 Estación Experimental de Aula Dei-CSIC, Avda de Montañana 1005, 50059 Zaragoza, Spain; vitemma@live.com (V.I.O.); jmontenegro@eead.csic.es (J.M.); saidanifaiza@hotmail.fr (F.S.); 2 Instituto Agroalimentario de Aragón IA2, CITA-Universidad de Zaragoza, 50013 Zaragoza, Spain; barriuso@unizar.es; 3 Centre Technique Interprofessionnel des Fruits et Légumes (Ctifl), route de Mollégès, F-13210 Saint-Rémy-de-Provence, France; aubert@ctifl.fr

**Keywords:** *Prunus persica*, *Monilinia*, genetic disease tolerance, ascorbic, phenolic acids

## Abstract

Brown rot, caused by *Monilinia* spp., provokes pre- and post-harvest damage in peach (*Prunus persica* (L.) Batsch), which causes an economic impact in the industry. With a view to breeding for increased tolerance to this disease, a screening test based upon artificial fruit inoculation was validated on several parental lines of a peach breeding program during the two-period harvest. In addition, cultivars with different total phenolic contents were included in the two-year study. All physicochemical fruit traits recorded at harvest showed differences among all cultivars. The antioxidant compound content determined using spectrophotometry (to measure ascorbic acid and antioxidant capacity) and UPLC-MS (to measure and identify phenolic compounds) also revealed important differences among all genotypes. The rate of brown rot lesion following fruit inoculation varied widely among cultivars, and it was possible to discriminate between highly and less susceptible cultivars. Cultivars with minimal development of damage were identified as germplasm with the desirable allele combination to increase brown rot tolerance in peach breeding programs. Finally, Pearson’s correlation coefficients (r) between pairs of variables were calculated, searching for any biochemical candidate conferring tolerance. The correlation of phytopathological traits with the antioxidant composition, concerning contents of ascorbic, neochlorogenic, and chlorogenic acids and total polyphenols in fruit, is discussed.

## 1. Introduction

One of the most important stone fruit crops in Spain is peaches (*Prunus persica* (L.) Batsch) with a production of 903,809 tons in 2018 [1], though this crop is often hindered by the activities of pathogenic fungi. The most important fungal disease agent of peaches and nectarines in Spain is *Monilinia* spp. The most extended species with the greatest degree of damage is *Monilinia laxa* (Aderhold and Ruhland) Honey, the occurrence of which is currently at the same relative frequency as *M. fructicola* (G. Winter) Honey [2,3,4,5]. Both species have been associated with about 85%–90% of brown rot (BR) incidence in Spanish peach commodities [2]. The pathogen can initiate infection in the flower and later develop in fruit during storage. In general, yield losses have been recorded, especially after harvest, reaching 80%–85% depending on the meteorological conditions [6,7], obviously posing a great threat for sustainable production of crops.

Common control practices for this fungal pathogen, especially in the Spanish orchards, were represented by the use of preventive and systemic fungicides such as thiophanate-methyl, iprodione, and cyproconazole [2]. However, the use of fungicides is becoming more limited because of consumer demands for residue-free fruit [8], including the fact that post-harvest treatments were limited by law until 2016 in Spain, and contamination of the environment should be avoided [9]. In addition, the steady increase of *Monilinia* strains resistant to fungicides, worldwide [9,10,11,12] and in Spain [2,13], has also been reported. All these adverse implications put together make it pertinent to search for alternatives with lasting and environmentally friendly effects, thus enhancing consumer acceptability [14,15]. In this direction, the availability of genotypes more tolerant and/or resistant to *Monilinia* spp. would be a safe solution in combination with a low rate of fungicide application and practical measures for sustainable peach production.

Nowadays, there is a considerable interest in the development of host-resistant peach cultivars and the identification of appropriate parameters related to resistance to *Monilinia* spp. One of the first studies identified high levels of brown rot resistance in the ”Bolinha” cultivar [16], and thereafter its tolerance was associated with the high content of phenolic compounds [17,18]. In peach, the influence of polyphenols has been well established [19] and widely studied in relation to brown rot tolerance [17,18,20,21,22,23,24]. The potential role of phenolic acids in combination with other factors in the resistance to the brown rot caused by *Monilinia* spp. has been discussed by several authors [5,25]. In particular in peach, it was described that chlorogenic and caffeic acids markedly inhibited the production of the cell wall degrading enzymes polygalacturonase and cutinase in *M. fructicola* cultures [21] but had no effect on *M. fructicola* growth [18]. Another study also pointed out that high doses of chlorogenic (CG) acid inhibits fungal melanin biosynthesis, which potentially diminishes *M. laxa* penetration [23]. However, most of the previous studies regarding phenolic acids have been conducted in vitro or in immature peach fruits, and no general conclusions can be derived concerning peach tolerance to brown rot. 

Furthermore, it would be interesting to test if other antioxidant compounds may prevent or reduce disease symptoms in peach fruits. In other plant species, it has been verified that exogenous applications of ascorbate, dehydroascorbate, or glutathione reduce disease lesions in infected Arabidopsis, *Solanum*, and *Nicotiana* plants [26,27,28]. To our knowledge, besides the phenolics, neither ascorbate nor glutathione or carotenoids have ever been considered in conferring tolerance to brown rot in peach. 

From a breeder point of view, it is noteworthy that although a great number of commercial stone fruit cultivars are susceptible to *Monilinia* spp. [5,29,30,31], there exists genetic control [32,33] that can be introgressed in the genetic background of high fruit quality cultivars [25]. Therefore, for breeding purposes, we evaluated eight commercial peach/nectarine cultivars for susceptibility to *M. laxa*. The objective of this study was to explore if the fruit antioxidant composition influences the development of brown rot damage after artificial inoculation. Here we discuss if the antioxidant composition such as endogenous ascorbic acid or NCG and CG phenolic acids in peach tissue may be involved in the host brown rot tolerance. 

## 2. Results

Peach fruits were harvested between June and October as ordered in Table 1. The earliest cultivars were “Crown Princess”, “Big Top”, and “Tebana” (mid-June to July). “Andross” and “Baby Gold 9” were harvested in August. In contrast, the Spanish non-melting flesh cultivars “Miraflores”, “Calanda Tardio”, and “Calante” were harvested the latest, from September to October. 

### 2.1. Physicochemical Parameters and Biochemical Composition 

All studied physicochemical parameters and biochemical contents of the cultivars at harvest are presented in Table 1. For all parameters, analysis of variance at 5% probability level showed significant differences among the cultivars. The pH values ranged from 3.65 in “Calante” to 4.31 in “Andross” (Table 1). The cultivars with pH ˃ 4 showed TA values below 0.5, and when pH was ˂4, TA values were over 0.5. The firmness ranged from 26.17 N in “Crown Princess” to 59.84 N in “Calanda Tardio”. Soluble solids content (SSC1) ranged from 10.58 to 15.53 °Brix in “Tebana” and “Calanda Tardio”, respectively. 

We analyzed the antioxidant composition in flesh fruit of the eight peach cultivars and determined the acid content, relative antioxidant capacity (RAC), and polyphenolic profile. The RAC, contents of ascorbic and the major hydroxycinnamic acids (neochlorogenic, NCG, and chlorogenic, CG), and total polyphenols (TPP) are presented in Table 1 and Tables S1 and S2. 

Ascorbic acid content was varied, ranging broadly from 4.09 in “Big Top” to 14.91 mg/100 g FW in “Andross”. The antioxidant activity ranged widely from “Big Top” to “Calanda Tardio” (RAC 20.01–186.45 mg TE/100 g FW). The nectarine “Big top” showed the lowest contents of ascorbic acid (AsA) and RAC although was not significantly different from “Crown Princess” and “Tebana”. In contrast, the cultivars “Calanda Tardio” followed by “Calante” showed the highest relative antioxidant capacity.

**Figure 1:**
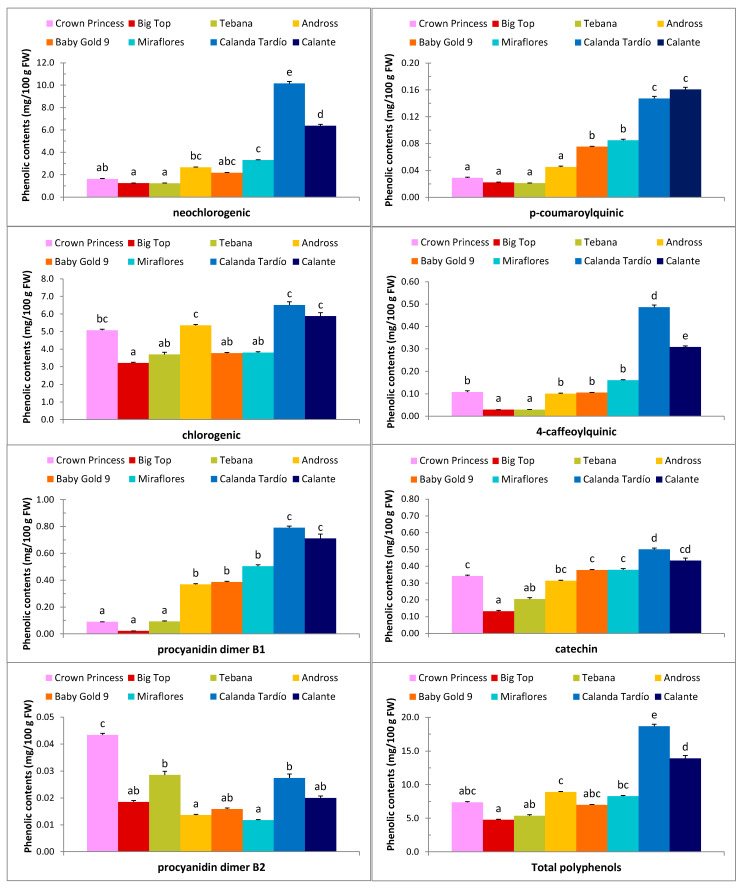
Composition of the main polyphenols in the fruit of eight peach cultivars harvested during 2014–2015. Data are means ± SE of N = 6 replications. Mean values with the same letter are not significantly different according to Duncan’s test at α = 0.05. See values in Supplementary Table S2.

Regarding polyphenols, significant variation was found among cultivars for contents of NCG and CG hydroxycinnamic acids and TPP (Table 1). Neochlorogenic acid (NCGA) content ranged from 1.24 to 1.25 mg/100 g FW in “Tebana” and “Big Top” to 10.16 mg/100 g FW in “Calanda Tardio”. Chlorogenic acid (CGA) ranged from 3.70 mg/100 g FW in “Big Top” and “Tebana” to 6.52 mg/100 g FW in “Calanda Tardio”. Figure 1 illustrates the polyphenolic compounds that were significantly different among the studied peach cultivars (hydroxycinnamic acids: neochlorogenic. p-coumaroylquinic, chlorogenic and 4-caffeoylquinic; flavanols: procyanidin dimer B1, catechin and procyanidin dimer B2; and total polyphenols). Hydroxycinnamic acids were the major polyphenolic compounds found in peach fruits (See Figure S1). Neochlorogenic and chlorogenic acids were the major hydroxycinnamic acids found in peach fruits (See Figure 1 and Table S2). Except for procyanidin dimer B2, in general the nectarine “Big Top” and “Tebana” showed the lowest levels of all phenolics. On the contrary, the late-maturing cultivars “Calanda Tardio” and “Calante” showed the highest levels of neochlorogenic, p-coumaroylquinic, chlorogenic and 4-caffeoylquinic, procyanidin dimer B1, catechin, and TPP. These two cultivars also showed the highest contents of total polyphenols (TPP), total hydroxycinnamic acids (HA), and total flavanols (FA) (Figure S1 and Table S2). 

### 2.2. Fruit Susceptibility 

**Figure 2:**
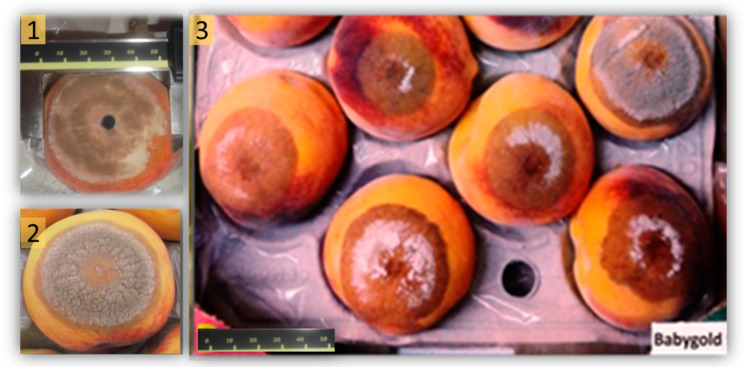
Different lesions caused by *Monilinia laxa* in different peach cultivars after five days of inoculation. (**1**) “Crown Princess”, (**2**) “Calante”, and (**3**) “Baby Gold 9”.

No visible symptoms of brown rot infection were observed on control mature fruits that had been inoculated with sterile distilled water and stored for five days. In mature fruits, symptoms of brown rot lesions appeared after three days of fruit inoculation and storage. Brown rot incidence (percentage of fruits with lesion), lesion diameter (LD = diameter of the lesioned fruit), and colonization extent (CE = diameter of the mycelium growth) were recorded as phytopathological parameters after fruit inoculation and storage (Figure 2). 

**Figure 3:**
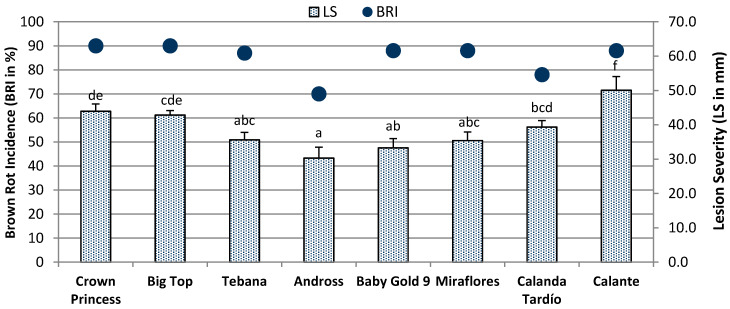
Brown rot incidence (%) and lesion severity (mm) on eight peach cultivars evaluated during two consecutive years (2014–2015). Data are means ± SE (N = 20–35 fruits). Different letters show significant differences of lesion severity among cultivars according to Duncan’s test at *α* = 0.05.

After five days, brown rot incidence ranged from 70% to 90% among the eight studied cultivars (Figure 3). The lesion severity (LS), which combines brown rot incidence (BRI) and LD (LS = BRI × LD), ranged from 30.29 to 50.07 mm, with “Andross” being the more tolerant cultivar and “Calante” the more susceptible. “Andross” exhibited the lowest LS, significantly different from “Crown Princess”, “Big Top”, “Calanda Tardío”, “and “Calante”. After incubation, fruit firmness and SSC were also recorded (Table S3). Fruit firmness after five days of incubation in control fruits (mean FF2: 33.5 N) decreased significantly in *M. laxa* inoculated fruits (mean FF3: 29.2 N); however, no significant differences were found in soluble solids content between non-inoculated (mean SSC2 = 14.0 °Brix) and inoculated fruits (mean SSC3 = 13.5 °Brix) (Table S3).

### 2.3. Pearson’s Correlation

**Figure 4:**
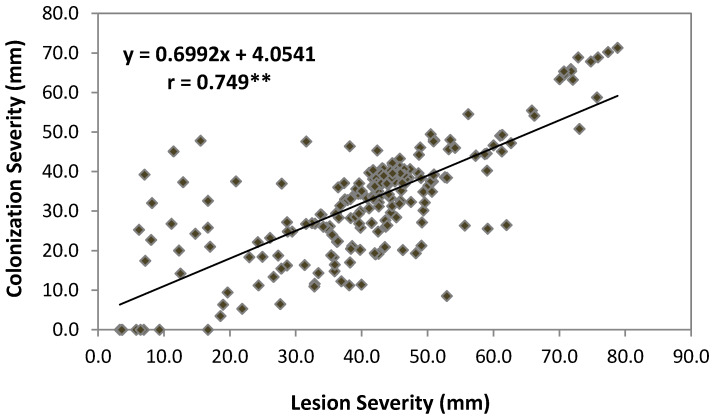
Linear regression between the lesion severity (%BRI × LD) with colonization severity (%C × CE) evaluated during two consecutive years (2014–2015) (N = 266 fruits). Significance of Pearson correlation is shown (** *p* < 0.01).

Pearson’s correlation coefficients (r) between pairs of phytopathological and antioxidant parameters were calculated with the purpose of highlighting their involvement in the host tolerance. All pathological traits (brown rot incidence, percentage of colonization, and lesion and colonization severities) were highly correlated among them (Table 2). Figure 4 shows the correlation between the lesion severity and the colonization severity (R^2^ = 0.562; r = 0.749, *p* ≤ 0.01).

In addition, the correlation analysis was carried out to clarify the contribution of the antioxidant content to *Monilinia* tolerance. The total antioxidant capacity (RAC) was highly correlated with phenolic compounds (NCG, CGA, and TPP), and all phenolics were also highly correlated among them (Table 2). The analysis between *M. laxa* and antioxidant parameters surprisingly revealed significant inverse correlation only with ascorbic acid content. Table 2 showed the inverse correlation between AsA content and lesion severity (r = −0.579, *p* ≤ 0.01) and colonization severity (r = −0.590, *p* ≤ 0.01). On the contrary, in this two-year study, no significant correlation was found between pathological traits and RAC or the polyphenolic compounds; neither the neochlorogenic nor the chlorogenic hydroxycinnamic acids or TPP were significantly correlated.

## 3. Discussion

In this research, we studied eight commercial peach cultivars with different fruit characteristics and tested their tolerance to brown rot caused by *Monilinia laxa* after artificial inoculation. As was reported in our previous study, symptoms of infection in peach fruits were only developed after inoculation upon commercial maturity [3,34]. A similar approach and protocols are routinely used to phenotype tolerance to brown rot caused by *M. fructicola* in other peach germplasm [35]. Based on the pH, these cultivars can be classified as acid (pH ˂ 4) or non-acid fruits (pH ˃ 4) [36]. However, the range of pH (3.7 to 4.3) found among cultivars has no effect on the in vivo *Monilinia* spp. growth as demonstrated by Obi and coworkers in a parallel experiment recording the in vitro mycelial growth at these pH ranges [34]. Values found for pH, TA, FF1, and SSC1 were within the range reported in other studies in peach germplasm [37,38,39]. The differences found among cultivars in firmness (FF1) are mainly related to factors such as harvest date or fruit type. Firmness of ripe peaches tended to be higher for the late cultivars, as was found in “Calanda Tardio” and “Calante” [40,41].

Similarly, the content for ascorbic acid varied widely (from 4.09 in “Big Top” to 14.91 mg/100 g FW in “Andross”) and fell within the ranges previously reported for ascorbic acid in peach pulp [37,38,39]. Regarding polyphenols, hydroxycinnamic acids were predominant in white-flesh cultivars as reported for other cultivars [42,43,44], the major NCG and CG acids being the highest. “Calanda Tardio” and “Calante” showed the highest contents of both hydroxycinnamic acids. These cultivars also showed the highest relative antioxidant capacity (RAC), total polyphenols (TPP), total hydroxycinnamic acids (HA), and total flavanols (FA) contents (Table 1, Table S2 and Figure S1), as reported in peel and flesh tissue in a parallel study [39]. The wide variation found among these cultivars has been attributed especially to climatic factors over the same crop season, although interaction was found between cultivar and year for some parameters (see Table S1). The cultivars that were harvested late in the season had high relative antioxidant capacity and contained higher contents of flavonoids and TPP than cultivars harvested earlier in the season [39]. The levels of antioxidants gradually increased as the harvest date progressed over the same crop season. This is consistent with the high and positive correlation (*p* ≤ 0.01) found between harvest date and relative antioxidant capacity (r = 0.836), total hydroxycinnamic acids (r = 0.742), total flavanols (r = 0.874), and total polyphenols (r = 0.761) [45]. 

Based on the results found here, the degree of susceptibility to brown rot on examined peach cultivars was not associated with the large differences in harvest dates. The cultivars were harvested from mid-June to early October, but no correlation was found between harvest date and brown rot tolerance, as was reported previously in other peach germplasm [3,46]. All pathological parameters (brown rot incidence, colonization, and lesion and colonization severities) were highly correlated among them [3,46]. After five days of incubation, significant decreases of fruit firmness were found in inoculated fruits (FF3 = 29.2 N vs. FF2 = 36.2 N) due to *Monilinia* activity [46]. However, no significant differences were found between the soluble solid contents of control fruits (SSC2 = 14.0 °Brix) and inoculated fruits (SSC3 = 13.5 °Brix) in agreement with previous results found in other progenies [46]. Therefore, there is no credible evidence that the activities of *Monilinia laxa* depleted soluble solid contents in the peach, as was discussed previously [46].

In this work, we show that only AsA content presented an inverse correlation with lesion severity (r = −0.555, *p* ≤ 0.01). On the contrary, none of the other bioactive compounds, the relative antioxidant capacity, or NCGA, CGA, or TPP were correlated with any of the disease parameters (Table 2). In agreement with these findings, no relation was detected between brown rot resistance to *M. fructicola* and concentration of phenolic compounds in Californian peach germplasm [17]. Apparently, phenolic compounds were not specifically involved in the cultivar tolerance to brown rot caused by *Monilia fructicola* or *M. laxa*. Nevertheless, we could suggest that a combination of different antioxidant compounds may confer partial immunity as was found in “Andross” [17]. This cultivar showed the highest level of ascorbic acid, high CG acid, and moderate relative antioxidant capacity, which may contribute to its tolerance. “Andross” also presented moderate levels of flavonoids and total phenolic content in peel and pulp tissues [39]. On the contrary, the levels of ascorbic, NCG, and CG acids found in “Calante” and the highest contents of RAC, NCGA, CGA, and TPP found in “Calanda Tardio” cannot explain their susceptibility to brown rot. 

As mentioned above, the role of plant phenolic acids in fungal inhibition has been widely discussed by several authors [5,24] (and references therein). CG and caffeic acids at levels similar to or in excess of those in the exocarp of immature resistant fruits did not affect *M. fructicola* growth [18]; however, these acids downregulate cutinase production in *M. fructicola* cultures [47] and markedly inhibit the production of the cell wall polygalacturonase and cutinase [21]. In the same direction, Villarino and coworkers [23] found that NCGA and CGA contents in immature fruits were negatively correlated to brown rot incidence and demonstrated in experiments in Petri dishes in vitro that high CGA concentration modified fungal melanin production that might interfere with *Monilinia laxa* penetration. These studies suggest that phenolic acids may suppress the cellular activities in fungal pathogens, which may be crucial for their growth and colonization on a host. However, in this study, the correlation analysis revealed that NCGA and CGA contents or total polyphenols in fruits had no effect on fungal lesion after artificial inoculation. In other words, contents of NCG and CG acids at harvest are probably independent and do not indicate cultivar tolerance. 

On the contrary, the ascorbic acid, which is considered one of the most important antioxidants in plant tissues, was negatively correlated with fungal growth after artificial inoculation (Table 2). Our results are in agreement with several authors [47] who demonstrated that other antioxidants such as glutathione and lipoic acid significantly attenuate cutinase production in *M. fructicola* and discuss that the effect of phenolics is due to a general antioxidant effect rather than a specific chemical interaction. Lee et al. in 2007 [21] also mentioned that phenolics in plant tissue may influence the antioxidant level in the pathogen and, as a consequence, the expression of genes associated with infection. The content of ascorbic acid in peach, until now considered as an important nutritional quality indicator, can be considered more relevant for breeding purposes for its antioxidant role conferring in vivo brown rot tolerance. Our results are consistent with those previously reported in transgenic potato plants with enhanced levels of cellular L-ascorbate that resulted in the reduction of the disease symptoms caused by the oomycete fungi *Phytophthora infestans* [48]. These transgenic potato plants that overproduced AsA by 1.6–2 folds showed reduced necrotic spots compared to the control leaf and exhibited tolerance to various abiotic stresses due to the enhanced ROS-scavenging activity. In the same vein, it was reported that mutant plants of Arabidopsis with different ascorbate levels were more tolerant to viruses or to *Alternaria brassicicola* infections [26,27]. These authors conclude that host ascorbate levels are important for limiting *Alternaria brassicicola* disease severity in Arabidopsis [26] and that the balance of ascorbate and glutathione may be the reason for alleviation of virus symptoms [27].

Interestingly, it has been described that exogenous applications of ascorbic acid may alleviate disease symptoms. In particular, exogenous application of AsA alone or in combination with other substances has an inhibitory growth effect on the pathogen *Alternaria brassicicola* in Arabidopsis [26], efficiently alleviates the damage caused by the *Phytoplasma* in infected potato tubers [28], and alleviates the symptoms and eventually inhibits RNA virus replication in leaves of Arabidopsis and *Nicotiana benthamiana* plants [27]. In virus-infected leaves, AsA at a high doses is able to inhibit virus replication and parrying symptoms [27]. These findings will open new research to test the effect of ascorbic acid either in vivo on brown rot tolerance or in vitro on microbial growth. We conclude that in the future, the contribution of single phenolic compounds or AsA to the antioxidant capacity and their relation to *Monilinia* spp. infection should be further investigated.

## 4. Materials and Methods

### 4.1. Peach Cultivars

The studied peach cultivars were maintained in an orchard located at the Experimental Station of Aula Dei-CSIC, Zaragoza, Spain (41°43′ N, 0°48′ W). Three trees per genotype were trained to the standard open vase system and planted at a spacing of 4 × 2.5 m and grown under standard conditions of irrigation, fertilization, and pest and disease control. In the two-year study (2014–2015), any fungicide treatment was applied in the field before harvest with adequate consideration to the free entry period for evaluation. The preventive fungicide Teldor^®^ 500 SC (fenhexamid) was sprayed at the Aula Dei Experimental Station (EEAD) on 29 July 2014, and 15 September 2015. All cultivars were non-melting-flesh peach, except the melting-flesh nectarine “Big Top”. All fruits were yellow-flesh, and their origin harvest dates and fruit characteristics are shown in Table 1. 

At optimal maturity, 25 fruits were harvested to evaluate tolerance, and 20 fruits were harvested to determine physicochemical parameters and biochemical analysis. For inoculation purposes, 25 mature fruits were harvested and disinfected by immersion in an aqueous solution of 1.6% sodium hypochlorite (commercial), 0.005% Tween^®^ 80 (polysorbate surfactant), and 1.6% ethyl alcohol for 4 minutes; rinsed in sterile distilled water; and spread out on sterile stone-fruit holding cardboard boxes for 20 minutes of ambient air drying in the blossom-stem (upside down) position to avoid any possible percolation at the stem position. After storage incubation, fruit firmness and SSC were also recorded in control (FF2, SSC2) and inoculated fruits (FF3, SSC3) to test the storage effect.

### 4.2. Physicochemical and Biochemical Quality Parameters 

At harvest, 20 fruits were hand picked out at commercial maturity to determine basic quality parameters (pH, titratable acidity (TA), fruit firmness (FF1), and soluble solids content (SSC1) [39]). Fruits were peeled, and flesh tissue was cut into small pieces (three replicates of five fruits each). For each replicate, five grams of flesh tissue were frozen in liquid nitrogen and stored at −20 or −80 °C for further biochemical analysis [39]. 

Contents of ascorbic acid, antioxidant capacity, and phenolic compounds were determined according to [39]. Ascorbic acid and antioxidant capacity were measured using spectrophotometry, and polyphenols, including the major hydroxycinnamic neochlorogenic (NCGA) and chlorogenic (CGA) acids, were identified using ultra performance liquid chromatography coupled with tandem mass spectrophotometry (UPLC-MS) and quantified using UPLC-diode-array detector. Briefly, the ascorbic acid was extracted with 10 mL of 5% HPO3. To determine the antioxidant capacity and total polyphenols, samples were extracted with 10 mL of a mixture of MeOH/H2O/formic acid (60:38:2 v/v/v). Ascorbic acid determination was based on the reduction of Fe (III) to Fe (II) by L-ascorbic acid followed by the formation of Fe (II) −2, 2′-Bipyridil complex [49]. Absorbance was measured at 525 nm, and the results are expressed as mg of ascorbic acid (AsA) per 100 g of FW following the calibration curve prepared daily. The antioxidant capacity was measured using the 1,1-diphenyl-2-picrylhydrazyl (DPPH) method [50]. The extract was mixed with DPPH for 60 min in darkness at room temperature. Absorbance was measured at 535 nm, and the results are expressed as relative antioxidant capacity (RAC) in mg of Trolox equivalents (TE) per 100 g of FW following the calibration curve prepared daily. For analysis of phenolics, one hundred micrograms of internal standard (methyl 4-hydroxybenzoate) was added to an aliquot of the polyphenol extract, which was concentrated to dryness with a speed vac at 45 °C. After the addition of 1 mL of the mixture of MeOH/formic acid (95:5 v/v), 1 μL of the solubilized polyphenolic extract was directly injected into the UPLC with a photodiode array detector (280, 330, and 520 nm), and UPLC-MS coupling was performed with a Bruker Daltonics HCT ultra ion trap mass spectrometer equipped with an electrospray ionization source [42].

### 4.3. In Vivo Assay: Inoculum, Inoculation, and Brown Rot Evaluation

The culturing of the *M. laxa* (Aderhold and Ruhland) Honey and the inoculation and the evaluation of brown rot tolerance was carried out according to the protocol described in [3]. The isolate (CPML02) was provided by the Collection of Postharvest Pathology Group of IRTA (Lleida) and was isolated from an infected peach fruit from a commercial orchard in the Cataluña region of Spain. 

**Figure 5:**
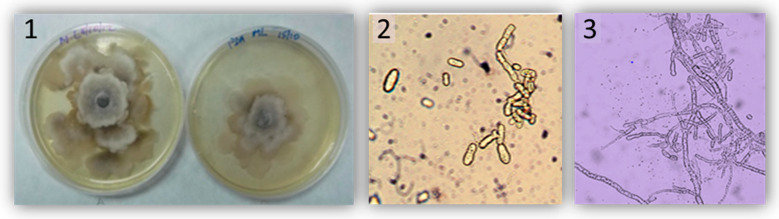
Infective propagules of *Monilinia laxa*: (**1**) Petri dishes with *M. laxa* growing in PDA media over 3–5 days, (**2**) conidia of *M. laxa* (400×), (**3**) mycelia of *M. laxa* (400×).

The strain was maintained at 4 °C in the dark on 39 gL^−1^ solid potato dextrose agar (PDA) media (Panreac, Spain) (Figure 5). The inoculation was conducted with 25 µL of a suspension of 25 conidia/µL of *M. laxa* on 20 fruits per cultivar, without skin injury. Five fruits per cultivar were used as control and inoculated with 25 µL of sterile water to discard unspecific infections. All fruits were incubated for five days at 23 °C and 45%–60% relative humidity. Brown rot incidence (%BRI), colonization (%C), lesion diameter (LD = diameter of the lesioned fruit), and colonization extent (CE = diameter of the mycelium growth) were the pathogenic parameters measured after 5 days (Figure 2). Lesion and colonization severities (LS and CS) in each cultivar were calculated as percentage of infected fruits x lesion diameter and percentage of colonized fruits x colonization extent, respectively [3,32,35]. Colonization (%C), colonization extent (CE mm), and colonization severity were recorded to test the correlations with lesion damage. 

### 4.4. Statistical Analysis

All statistical analysis was performed using SPSS 26.0 (SPSS Inc.; Chicago, IL, USA). For biochemical determinations, three biological replicates were considered for the years of analysis (2014–2015); mean and standard error (SE) were calculated for each parameter. The phytopathological parameters were recorded for each cultivar in 5 individual fruits inoculated with water for control and 20 individual fruits inoculated with *M. laxa* spores for the years of analysis (2014–2015); mean and standard error (SE) were calculated for each parameter. Two-way ANOVA was performed for main effects (cultivar and year) and their interaction (cultivar × year), and mean comparison was judged according to Duncan’s test at the level *p* ≤ 0.05. Pearson’s correlation and regression analysis (Microsoft Excel 10.0) was conducted to reveal possible association between pairs of parameters.

## 5. Conclusions

In conclusion, the result of the evaluation of tolerance in the eight commercial cultivars to brown rot disease demonstrates variability in the susceptibility to *Monilinia laxa*, with “Andross” and “Baby Gold 9” the more tolerant but not significantly different from “Tebana” and “Miraflores”. “Crown Princess”, “Big Top”, “Calante”, and “Calanda Tardío” were the less tolerant peach cultivars. The content of ascorbic acid can only partially explain the tolerance/susceptibility observed, indicating that other factors are probably involved in the response. Identification of these factors will be fundamental for breeding programs focused on improving resistance. In addition, neither neochlorogenic nor chlorogenic acids or TPP contents at harvest can explain the differences in tolerance to brown rot found in these peach cultivars. We suggest that together with the ascorbic acid, other physicochemical factors may confer the tolerance to “Andross”, “Tebana”, “Baby Gold 9”, and “Miraflores”. To our knowledge, this is the first study that correlates the ascorbic acid contents in mature peach fruit with *Monilinia laxa* brown rot tolerance. From a practical point of view, application of ascorbate based formulae may be promising to decrease the damage caused by *Monilinia laxa*. Furthermore, we speculate that the complex *Monilinia–Prunus* interaction establishes the redox environment that modulates or restricts the fungal infection. Nonetheless, further studies need to be done in order to determine the effect of AsA as a curative or preventive treatment to control *M. laxa* infections and/or to disentangle the role of each specific antioxidant compound in brown rot tolerance in peach.

## Figures and Tables

**Table 1:** Characteristics of the eight peach cultivars: Cultivar name, origin, harvest dates and fruit physicochemical parameters. Data are means of two years (2014–2015). Contents of ascorbic acid, RAC, NCGA, CGA, and TPP in the pulp (data are means of three replications for two years, N = 6).

Cultivars	Origin	Harvest Date	pH	TA	FF1	SSC1	AsA*	RAC*	NCGA*	CGA*	TPP*
					(N)	(°Brix)					
Crown Princess	USA	19-June a	3.82 a	0.56 b	26.17 a	10.85 a	5.18 a	54.60 bc	1.63 ab	5.08 bc	7.37 abc
Big Top	USA	03-July b	4.15 b	0.42 a	31.95 b	12.25 ab	4.09 a	20.01 a	1.25 a	3.21 a	4.77 a
Tebana	Italy	03-July b	4.12 b	0.43 a	33.04 bc	10.58 a	9.11 bc	38.43 ab	1.24 a	3.70 ab	5.37 ab
Andross	USA	02-Aug c	4.31 b	0.35 a	31.56 b	14.15 bc	14.91 d	88.08 d	2.66 bc	5.36 c	8.91 c
Baby Gold 9	USA	21-Aug d	4.17 b	0.41 a	36.41 b	13.68 bc	8.75 b	75.36 cd	2.17 abc	3.77 ab	6.99 abc
Miraflores	Spain	10-Sep e	3.79 a	0.61 b	35.49 bc	13.43 bc	10.88 c	89.82 d	3.31 c	3.80 ab	8.28 bc
Calanda Tardío	Spain	07-Oct f	3.68 a	0.75 c	59.84 e	15.53 c	8.89 b	186.45 e	10.16 e	6.52 c	18.67 e
Calante	Spain	07-Oct f	3.65 a	0.72 c	46.18 d	14.55 bc	10.72 bc	168.63 e	6.37 d	5.88 c	13.92 d

Abbreviations and units: Titratable Acidity (TA) = g/100 g; fruit firmness at harvest (FF1) = Newton (N); soluble solids content at harvest (SSC1); ascorbic acid (AsA) = mg AsA/100 g fresh weight, relative antioxidant capacity (RAC) = mg Trolox equivalent/100 g FW; neochlorogenic acid (NCGA) = mg/100 g FW, chlorogenic acid (CGA) = mg/100 g FW; total polyphenols (TPP) = mg/100 g FW. * Data are means of (N = 6). For each column, mean values with the same letter are not significantly different according to Duncan’s test at *α* = 0.05.

**Table 2:** Pearson’s correlation coefficients between pathological and antioxidant parameters in eight peach cultivars harvested over two years (2014–2015). N = 37–319.

Trait	LS	%C	CS	AsA	RAC	NCGA	CGA	TPP
**%BRI**	0.471**	0.832**	0.682**	−0.537**	ns	ns	ns	ns
**LS**		0.597**	0.749**	−0.579**	ns	ns	ns	ns
**%C**			0.830**	−0.652**	ns	ns	ns	ns
**CS**				−0.590**	ns	ns	ns	ns
AsA					ns	ns	ns	ns
RAC						0.876**	0.704**	0.967**
NCGA							0.707**	0.970**
CGA								0.861**

Significance: ** *p* ≤ 0.01. Units and abbreviations: %BRI: percentage of brown rot incidence; LS: lesion severity (mm); %C: percentage of colonization; CS: colonization severity (mm); ascorbic acid (AsA) = mg AsA/100 g FW; relative antioxidant capacity (RAC) = mg Trolox equivalent/100 g FW; neochlorogenic acid (NCGA), chlorogenic acid (CGA), and total polyphenols (TPP) in mg/100 g FW. Negative correlations are in bold.

## Supplementary Materials

The following are available online at https://www.mdpi.com/2223-7747/9/5/589/s1, Figure S1: Total polyphenolic composition in pulp of eight peach cultivars harvested during 2014–2015. Table S1: Ascorbic acid, relative antioxidant activity, neochlorogenic, chlorogenic and total polyphenols contents in pulp of eight peach cultivars harvested in 2014–2015. Data are mean of N = 3 replications per year, Table S2: Polyphenolic compounds content (mg/100 g FW) in pulp of eight peach cultivars harvested during two years (2014–2015). Data are mean of N = 3 replications per year, Table S3: Brown rot incidence, lesion severity, fruit firmness and solid solids content in eight peach cultivars after five days of storage. Data are mean ± SE (N = 5–20 fruits) for two consecutive years (2014–2015).
