# Supplementary material for: Is the Tolerance of Commercial Peach Cultivars to Brown Rot Caused by Monilinia laxa Modulated by its Antioxidant Content?
Source: Plants (Basel). 2020 May 5;9(5):589. doi: 10.3390/plants9050589 (PMC7285238; doi:10.3390/plants9050589)
Supplement: Supplementary file 1 [file plants-09-00589-s001.pdf]

Supplementary Table 1. Ascorbic acid, relative antioxidant activity, neochlorogenic, chlorogenic and total polyphenols contents in pulp of eight peach cultivars harvested in 2014-2015. Data are mean of N=3 replications per year.

**Cultivars**	**Year**	Ascorbic acid	RAC	NCGA	CGA	TPP
	2014	9.17	98.10 b	3.58	4.96	9.62
	2015	9.23	82.25 a	3.61	4.37	8.95
	**Signification**	ns	**	ns	ns	ns
Crown Princess	2014	5.09 ab	53.00 b	1.11	4.73 bcdef	6.39
	2015	5.27 b	56.20 b	2.14	5.43 defg	8.35
Big Top	2014	4.02 a	16.77 a	1.28	3.04 ab	4.62
	2015	4.15 ab	23.25 a	1.21	3.38 abc	4.92
Tebana	2014	9.10 d	54.15 b	1.46	5.05 cdef	7.12
	2015	9.12 d	22.7 a	1.02	2.35 a	3.62
Andross	2014	17.34 h	95.24 c	2.69	5.62 efg	9.24
	2015	12.48 g	80.93 bc	2.63	5.09 cdef	8.57
Baby Gold 9	2014	9.88 de	73.89 bc	2.08	3.52 abcd	6.63
	2015	7.62 c	76.82 bc	2.27	4.02 abcde	7.34
Miraflores	2014	10.31 ef	100.67 cd	3.24	4.24 abcde	8.78
	2015	11.44 fg	78.98 bc	3.37	3.36 abc	7.79
Calanda Tardio	2014	7.39 c	178.80 e	9.58	6.42 fg	17.98
	2015	10.40 ef	194.10 ef	10.73	6.61 fg	19.37
Calante	2014	8.74 d	212.25 f	7.21	7.05 g	16.22
	2015	12.04 g	125.00 d	5.54	4.71 bcdef	11.63
**Interaction**	**Signification**	***	***	NS	*	NS

Two-way ANOVA was performed for lineal model on raw data followed by Duncan's test. Significance: at *P≤0.05, ***P≤0.001, and NS indicates not significant. Values in the same column followed by different letters were significantly different at P≤0.05, ascorbic acid (AsA) = mg AsA/100 g FW, antioxidant capacity (RAC) = mg TE/100 g FW, trolox equivalents (TE), neochlorogenic acid (NCGA), chlorogenic acid (CGA) and total polyphenols content (TPP) = mg /100 g FW.

Supplementary Table 2. Polyphenolic compounds content (mg/100 g FW) in pulp of eight peach cultivars harvested during two years (2014-2015). Data are mean of N=3 replications per year.

	Hydroxycinnamic acids						Flavanols					
	HA1	HA2	HA3	HA4	HA5	HA	FA1	FA2	FA3	FA	ANT1	TPP
**Year**												
2014	3.58	0.08 b	4.96	0.15 a	0.04	8.81	0.42 b	0.36	0.02	0.80 b	0.01 a	9.62
2015	3.61	0.07 a	4.37	0.18 b	0.04	8.28	0.32 a	0.31	0.02	0.65 a	0.02 b	8.95
**Cultivar**												
Crown Princess	1.63 ab	0.03 a	5.08 bc	0.11 b	0.04	6.89 abc	0.09 a	0.34 c	0.04 c	0.47 ab	0.01 a	7.37 abc
Big Top	1.25 a	0.02 a	3.21 a	0.03 a	0.04	4.55 a	0.02 a	0.13 a	0.02 ab	0.17 a	0.05 b	4.77 a
Tebana	1.24 a	0.02 a	3.70 ab	0.03 a	0.05	5.04 ab	0.09 a	0.20 ab	0.03 b	0.33 a	<0.01a	5.37 ab
Andross	2.66 bc	0.05 a	5.36 c	0.10 b	0.05	8.21 c	0.37 b	0.31 bc	0.01 a	0.70 bc	nd	8.91 c
Baby Gold 9	2.17 abc	0.08 b	3.77 ab	0.11 b	0.04	6.16 abc	0.39 b	0.38 c	0.02 ab	0.78 c	0.04 b	6.99 abc
Miraflores	3.31 c	0.09 b	3.80 ab	0.16 b	0.03	7.39 bc	0.50 b	0.38 c	0.01 a	0.89 cd	<0.01a	8.28 bc
Calanda Tardio	10.16 e	0.15 c	6.52 c	0.49 d	0.05	17.35 e	0.79 c	0.50 d	0.03 b	1.32 e	nd	18.67 e
Calante	6.37 d	0.16 c	5.88 c	0.31 c	0.04	12.76 d	0.71 c	0.43 cd	0.02 ab	1.16 de	nd	13.92 d
**Signification**												
Year	NS	*	NS	**	NS	NS	*	NS	NS	*	**	NS
Cultivar	***	***	***	***	NS	***	***	***	***	***	***	***
Year*Cultivar	NS	NS	*	**	NS	NS	NS	NS	***	NS	***	NS

Two-way ANOVA was performed for lineal model on raw data followed by Duncan test (P<0.05). Values in the same column followed by different letters were significantly different. Significance: *P≤0.05, **P≤0.01,***P≤0.001 and NS indicates not significant. Abbreviations: not detectable (nd), Neochlorogenic acid (HA1), p-coumaroylquinic acid (HA2), chlorogenic acid (HA3), 4-caffeoylquinic acid (HA4), caffeoylquinic acid derivative (HA5), Total hydroxycinnamic acids (HA), procyanidin dimer B1(FA1), (+)-catechin (FA2), procyanidin dimer B2 (FA3), Total flavanols (FA), cyanidin-3-glucoside (ANT1), Total polyphenols (TPP), non-detected (nd).

Supplementary Table 3. Brown rot incidence, lesion severity, fruit firmness and solid solids content in eight peach cultivars after five days of storage. Data are mean ± SE (N=5-20 fruits) for two consecutive years (2014-2015).

**Cultivars**	**BRI (%)**	**LS ± SE (mm)**	**FF2 (N)**	**FF3 (N)**	**SSC2 (°Brix)**	**SSC3 (°Brix)**
Crown Princess	90	43.94 ± 2.1 de	38.47 c	30.73 d	10.51 a	10.14 a
Big Top	90	42.82 ± 1.3 cde	18.33 a	12.12 a	15.05 bc	15.78 d
Tebana	87	35.62 ± 2.2 abc	28.62 b	28.65 cd	11.68 a	10.57 a
Andross	70	30.29 ± 3.2 a	26.22 b	24.51 b	14.55 bc	14.12 c
Baby Gold 9	88	33.29 ± 2.7 ab	30.77 b	27.14 bc	14.58 bc	13.07 b
Miraflores	88	35.39 ± 2.5 abc	27.15 b	26.53 bc	14.14 b	14.14 c
Calanda Tardío	78	39.33 ± 1.9 bcd	51.05 d	43.29 f	16.40 c	16.26 d
Calante	88	50.07 ± 4.0 f	48.12 d	40.46 e	14.48 bc	13.07 b

Abbreviations: BRI: brown rot incidence; LS: lesion severity; FF2: fruit firmness in non-inoculated fruits; FF3: fruit firmness in inoculated fruits; SSC2 and SSC3: soluble solids content in inoculated and non-inoculated fruits. N: newton; SE: standard error. For each column, mean values with the same letter are not significantly different at $p < 0.05$ (Duncan test).

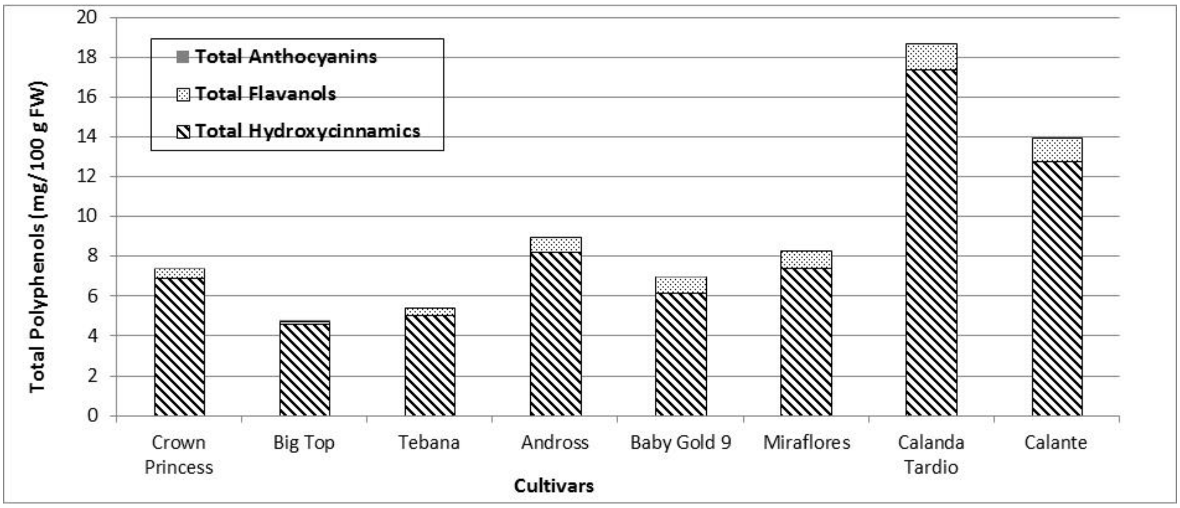

Figure S1. Total polyphenolic composition in pulp of eight peach cultivars harvested during 2014-2015. See contents in Supplementary Table 1.
